# Interleukin-4 Regulates Eomesodermin in CD8^+^ T Cell Development and Differentiation

**DOI:** 10.1371/journal.pone.0106659

**Published:** 2014-09-10

**Authors:** Shannon A. Carty, Gary A. Koretzky, Martha S. Jordan

**Affiliations:** 1 Department of Medicine, Perelman School of Medicine, University of Pennsylvania, Philadelphia, Pennsylvania, United States of America; 2 Abramson Family Cancer Research Institute, Perelman School of Medicine, University of Pennsylvania, Philadelphia, Pennsylvania, United States of America; 3 Department of Pathology and Laboratory Medicine, Perelman School of Medicine, University of Pennsylvania, Philadelphia, Pennsylvania, United States of America; Institut Pasteur, France

## Abstract

Interleukin (IL)-4 is a cytokine classically associated with CD4^+^ T helper type 2 differentiation, but has been recently shown to also be required for the development of CD8^+^ innate-like lymphocytes. CD8^+^ innate-like lymphocytes are non-conventional lymphocytes that exhibit characteristics typically associated with memory CD8^+^ T cells, including expression of the T-box transcription factor Eomesodermin (Eomes). Here we investigate the signaling pathways required for IL-4 induction of Eomes and CD8^+^ innate-like lymphocyte markers in murine CD8SP thymocytes and peripheral CD8^+^ T cells. We demonstrate that IL-4 is sufficient to drive Eomes expression and the CD8^+^ innate-like lymphocyte phenotype through cooperation between STAT6- and Akt-dependent pathways. Furthermore, we show that while IL-4 has little effect on the induction of Eomes in the setting of robust T cell receptor (TCR) activation, this cytokine promotes Eomes in the setting of attenuated TCR stimulation in mature CD8^+^ T cells suggesting that cytokine signaling pathways may direct cell fate when TCR signals are limiting.

## Introduction

Cytokines regulate T cell development and function [1],[2]. Interleukin (IL)-4 is a common γ-chain cytokine, known to regulate CD4^+^ T helper (T_H_) cell differentiation [3]. It has been shown to promote differentiation of naïve CD4^+^ T cells into the T_H_2 subset, which is essential for immunity to extracellular parasites, and to inhibit IFNγ production and T_H_1 responses [4], [5]. In addition, it has been implicated in allergic responses and asthma [6], [7]. Although IL-4 is classically associated with CD4^+^ T_H_2 differentiation and associated immune responses, it is also important in regulating CD8^+^ T cell responses during bacterial and parasitic infections [8], [9] and more recently has been demonstrated to be required for the development of a population of CD8^+^ innate-like lymphocytes (ILLs) [10]–[15].

ILLs are a diverse set of non-conventional T lymphocytes that develop in the thymus along with conventional T cells; however, unlike conventional T cells that require peripheral activation to develop effector function, ILLs acquire surface expression of activation/memory markers and effector function during development. ILLs include invariant natural killer T (iNKT) cells, γδ T cells and several CD8^+^ subsets, including H2-M3 restricted T cells, mucosal invariant T cells and CD8αα T cells [16]. CD8^+^ ILLs that are induced during development following exposure to IL-4 express high levels of CD44, CD122 (the β chain of the IL-2 and IL-15 receptors), IL-4 receptor alpha (IL4Ra) and CXCR3. They are primed for rapid IFNγ production upon *ex vivo* stimulation and are characterized by abundant expression of Eomesodermin (Eomes), a T-box transcription factor important for regulating CD8^+^ T effector cell and memory cell fate and function [17], [18]. This class of CD8^+^ ILLs has also been shown to possess enhanced function *in vivo*, as they produce more IFNγ following stimulation with either T cell receptor (TCR) or inflammatory cytokines and provide better protection against *Listeria monocytogenes* compared to naïve CD8^+^ T cells [11], [19], [20].

CD8^+^ ILLs are present in wild-type (WT) mice [12]–[14], [20], [21] and humans [14]; however, much of what we know about their developmental requirements has been learned through studies in mutant mice where this population is dramatically expanded. Such models include mice bearing mutations in or deficiencies of specific molecules downstream of the TCR (e.g. Itk, SLP-76) or transcriptional regulators (e.g. Id3, KLF2) [12], [21]–[25]. A shared feature of these systems is an increase in the number of promyelocytic leukemia zinc finger (PLZF)^+^ cells capable of producing IL-4 within the thymus. IL-4 from these cells acts in a cell-extrinsic manner on developing thymocytes to promote Eomes expression and CD8^+^ ILL development. While both IL-4 and Eomes have been shown to be required for CD8^+^ ILL development [12], the signals downstream of the IL-4 receptor that are responsible for directing expression of Eomes and other CD8^+^ ILL markers in thymocytes have not been defined fully.

IL-4 can also influence the function of mature CD8^+^ T cells. Early work suggested that IL-4 may promote anti-tumor effects of CD8^+^ tumor-infiltrating lymphocytes and promote persistence of CD8^+^ T cells [26], [27]. In addition, during malarial infection, IL-4 is required for the generation of protective CD8^+^ memory T cells [8]. Inhibition of proximal TCR signaling molecules has been shown more recently to allow IL-4 to promote Eomes expression in naïve CD8^+^ T cells undergoing TCR activation [28].

Here we investigate the signaling pathways responsible for IL-4-induced Eomes expression in CD8 single-positive (SP; CD8^+^CD4^−^) thymocytes and peripheral CD8^+^ T cells. We find that IL-4 is sufficient to promote Eomes expression and aspects of the CD8^+^ ILL phenotype via Akt and STAT6 signaling pathways. We also demonstrate that IL-4 and TCR stimulus synergize to promote IFNγ expression in activated CD8^+^ T cells, but IL-4 preferentially induces Eomes expression in peripheral CD8^+^ T cells exposed to low dose TCR stimulus.

## Materials and Methods

### Mice

SLP-76 Y145F mice have been described previously [29]. B6.PL-*Thy1^a^*/CyJ (Thy1.1^+^), B6.129S2(C)-*STAT6^tm1Gru^*/J mice were purchased from Jackson Laboratories. C57BL/6J mice were obtained from Jackson Laboratories and also bred in the University of Pennsylvania facility. Animals were housed at the University of Pennsylvania.

### Ethics Statement

These experiments were carried out in strict accordance with the recommendations in the Guide for the Care and Use of Animals of the National Institutes of Health and approved by the Institutional Animal Care and Use Committee (IACUC) of the University of Pennsylvania (protocol #803976).

### Antibodies and reagents for flow cytometry

The following antibodies were used (from BD Biosciences unless otherwise noted): CD8α-Pacific Blue or AlexaFluor (AF)700 (53-6.7, Biolegend); CD4 fluoroscein isothiocyanate (FITC) (GK1.5, eBiosciences), allophycocyanin (APC) (RM4-5), phycoerythrin (PE)-Cy5 or PE-Cy7 (RM4-5, Biolegend), or PE-TexasRed (RM4-5, Invitrogen), TCRβ APC-e780 (H57-597, eBiosciences), IL4Ra PE (mIL4R-M1), CD62L APC (MEL-14),, CD44 AF700 (IM7, Biolegend), CD122-biotin (TM-1) followed by streptavidin-PE Texas Red, Eomes-PE or AF647 (Dan11mag, eBiosciences), IFNγ-PerCPCy5.5 (XMG1.2, Biolegend), APC or PE, pSTAT6 PE (J1-773.58.11), and pAkt T308 PE (J1-223.371). For pS6 staining, cells were stained with a rabbit anti-phospoS6 monoclonal (2F9; Cell Signaling) and then stained with AF488 goat anti-rabbit IgG (Invitrogen). Live/Dead Aqua (Invitrogen) was used to exclude dead cells.

### Flow cytometry and cell sorting

Cells were isolated, washed and surface stained in phosphate-buffered saline (PBS) containing 2% fetal bovine serum for 30 min followed by additional washes. Intracellular staining was performed using either the Cytofix/Cytoperm kit (BD Biosciences) or FoxP3/Transcription Factor Staining Buffer kit (eBiosciences) according to manufacturer’s instructions. For phospho-flow cytometry, cells were fixed with pre-warmed BD Phosflow Lyse/Fix buffer, surface stained and then permeabalized with BD Perm/Wash Buffer prior to intracellular staining. Data were acquired using FACS LSR II (BD Biosciences) and analyzed with FlowJo software (TreeStar).

For cell sorting, CD8SP thymocytes were isolated after culture under indicated conditions by sorting of CD8^+^CD4^−^TCRb^+^ population on a FACS Arial II (BD Biosciences). CD8^+^ T cells from the spleen and lymph nodes were purified by negative selection and magnetic separation (CD8α^+^ T cell Isolation Kit II or Pan T cell isolation kit II; Miltenyi Biotec) followed by sorting of naïve CD8^+^ T cells (CD8^+^ CD4^–^CD44^–^CD62L^+^).

For experiments involving *ex vivo* stimulation, lymphocytes were stimulated with 500 ng/ml ionomycin and 50 ng/ml phorbol-12-myristate-13-acetate (PMA) in the presence of 1 mg/ml brefeldin A for 5 h and then analyzed by flow cytometry for intracellular cytokine staining.

### Thymocyte culture and fetal thymic organ culture

Thymocytes were cultured in T cell media (10% FCS, 50 µM 2-mercaptoethanol, 2 mM L-glutamine/penicillin/streptomycin in IMDM) alone or with murine IL-4 (20 ng/ml; Peprotech) for 20 h. For fetal thymic organ culture (FTOC), fetal thymic lobes were dissected from embryonic day (E) 14.5–15.5 embryos and cultured on sponge-supported filter membranes (sterile Gelfoam absorbable gelatin sponge, USP, 7 mm [Pfizer]; Nucleopore track-etched membranes, 0.8 µm pore-13 mm round [Whatman]) at the interphase between T cell media and 5% CO_2_-humidified air. Media was changed every 3–4 days of culture. Peripheral T cell populations were cultured in T cell media alone or in the presence of murine IL-4 (20 ng/ml) for 20 h. For experiments using inhibitors, 5 µM AKTi (Akt inhibitor VIII, Calbiochem) or 25 nM rapamycin (Calbiochem) was added and incubated for the indicated time.

### In vitro CD8^+^ T cell activation

Naïve CD8^+^ T cells were isolated via cell sorting and activated for 3d with plate-bound anti-CD3 (2C11, eBiosciences) at indicated doses and 5 µg/ml anti-CD28 (eBiosciences), followed by 2d of rest in T cell media supplemented with 0.5 ng/ml IL-2 (Peprotech).

### Real-time PCR

CD8SP thymocyte populations were isolated using cell sorting as indicated above. Cells were lysed in RLT buffer and total RNA was isolated using RNeasy Mini Kit (Qiagen) according to manufacturer’s instructions and cDNA was synthesized using Super Script III First Strand (Invitrogen). Real-time PCR was performed with primers specific for murine Eomes (Mm01351985_m1, Applied Biosystems) and β-actin (Mm_00607939_s1, Applied Biosystems) with Taqman Fast Universal Master Mix (Applied Biosystems) on a ViiA7 Real Time PCR system (Applied Biosystems). All samples were assayed in triplicate. For analysis, samples were normalized to internal β-actin levels and then compared to indicated control population by the relative quantification (ΔΔCT) method.

### Statistical analysis

Statistical significance was calculated using Student’s t-test or one-way ANOVA with Tukey’s post-test. Prism (GraphPad Software) was used for statistical analysis.

## Results

### IL-4 promotes Eomes expression in developing CD8SP thymocytes

**Figure 1:**
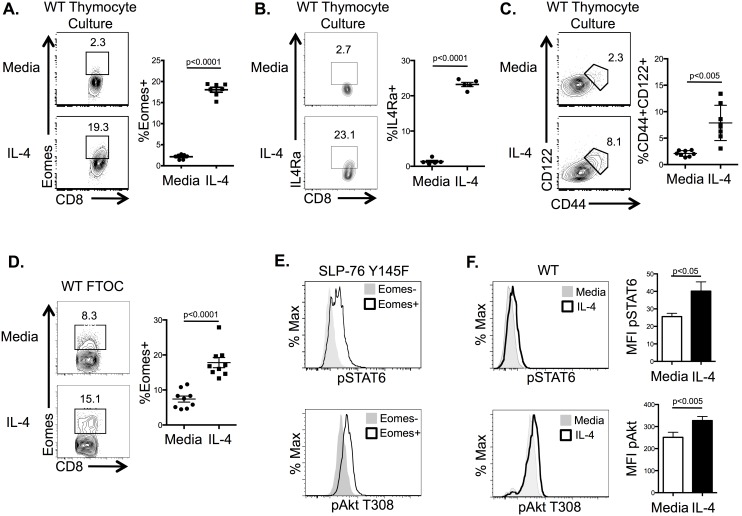
IL-4 promotes Eomes expression and CD8^+^ ILL development. A) Flow cytometric analysis of intracellular Eomes expression in CD8SP cells from WT thymocytes cultured in the absence or presence of IL-4 (20 ng/ml) for 20 h. Plots are gated on live, TCRβ^+^ CD8SP lymphocytes. *Right*, Percentage of Eomes^+^ cells among total CD8SP thymocytes in indicated conditions is shown (n = 7, 3 independent experiments). B) Representative flow cytometric analysis of IL4Ra on CD8SP cells from WT thymocytes cultured as above. *Right*, percentage of IL4Ra^+^ cells among total CD8SP thymocytes in indicated conditions (n = 5, 2 independent experiments). C) Flow cytometric analysis of surface CD44 and CD122 expression on CD8SP thymocytes treated under indicated conditions as above. *Right*, percentage of CD44^+^CD122^+^ cells among total CD8SP thymocytes (n = 8, 3 independent experiments). D) Flow cytometric analysis of intracellular Eomes expression in CD8SP thymocytes from d8 WT FTOC treated under indicated conditions. *Right*, percentage of Eomes^+^ cells among total CD8SP thymocytes in indicated conditions (n = 9, 2 independent experiments). E) Flow cytometric analysis of intracellular expression of phopsho-STAT6 (pSTAT6) and phospho-Akt T308 (pAkt) in Eomes^+^ CD8^+^ ILLs versus Eomes^−^ CD8SP thymocytes directly *ex vivo* from SLP-76 Y145F mice (n = 2–5). F) Flow cytometric analysis of intracellular expression of pSTAT6 and pAkt in TCRβ^+^ CD8SP thymocytes cultured with or without IL-4 *in vitro* for 20 h. *Right*, mean fluorescence intensity (MFI) of pSTAT6 and pAkt T308 under indicated conditions (n = 5, 2 independent experiments). Flow plots are gated on live, TCRβ^+^ CD8SP lymphocytes. Numbers in flow plots (A–D) represent the percent of the gated population. Graphs (A, B, C and D) show the average percentage of the indicated population and standard error of mean. Statistical significance calculated using Student’s t-test.

**Figure 2:**
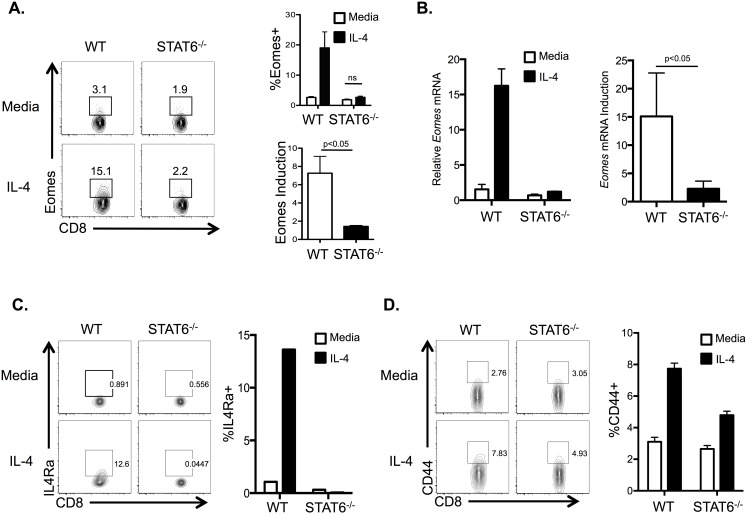
STAT6 is required for IL-4 regulation of Eomes in CD8SP thymocytes. A) Flow cytometric analysis of Eomes expression in WT and STAT6^−/−^ TCRβ^+^ CD8SP thymocytes after culture with or without IL-4 for 20 h. *Right top*, percentage of Eomes^+^ thymocytes among total CD8SP cells. *Right lower*, quantification of fold induction of Eomes in IL-4-treated CD8SP thymocytes of indicated genotypes. All data are representative of n = 3–4/genotype from 2 independent experiments. B) *Left*, relative Eomes expression in cDNA isolated from sorted CD8SP thymocytes in WT and STAT6^−/−^ thymocytes cultured in the absence or presence of IL-4 for 20 h, relative to WT CD8SP thymocyte population treated in media alone. *Right*, quantification of fold induction of Eomes expression in cDNA isolated from IL-4-treated CD8SP thymocytes of indicated genotypes, normalized to matched samples treated with media alone. Data are representative of n = 5/genotype, 2 independent experiments. C) Flow cytometric analysis of IL4Ra on CD8SP cells from WT thymocytes cultured as above. *Right*, percentage of IL4Ra^+^ cells among total CD8SP thymocytes in indicated conditions (n = 5/genotype, 2 independent experiments). D) Flow cytometric analysis of surface CD44 expression on CD8SP thymocytes treated under indicated conditions as above. *Right*, percentage of CD44^+^ cells among total CD8SP thymocytes (n = 5/genotype, 2 independent experiments). Numbers in flow plots (A, C, D) represent the percent of the gated population. Graphs show the average percentage (A, C, D) or fold induction (A, B) of the indicated population and standard error of mean. Statistical significance calculated using Student’s t-test.

IL-4 is required for *in vivo* CD8^+^ ILL development [11], [13]–[15], [25], [30] and *in vitro* treatment of WT thymocytes with IL-4 was sufficient to induce Eomes protein expression in CD8SP thymocytes (Figure 1A) and mRNA (Figure 2B, 3B). Specificity of Eomes staining was confirmed with an isotype control antibody (Figure S1). IL-4 also induced the expression of several CD8^+^ ILL markers in WT CD8SP thymocytes, including IL4Ra (Figure 1B), CD44 and CD122 (Figure 1C) in WT CD8SP thymocytes. However, CXCR3, a marker commonly associated with CD8^+^ ILLs was not upregulated under these conditions (data not shown), which may suggest that other signals are required for a full CD8^+^ ILL phenotype. The effect of IL-4 on CD8SP thymocyte development was also seen in fetal thymic organ culture (FTOC), which more closely mimics *in vivo* thymocyte development [31]. When WT fetal thymi were cultured in the presence of IL-4 for 8d, there was an increase in the percentage of developing CD8SP thymocytes expressing Eomes (Figure 1D). Consistent with a previous report [13], these data suggest that IL-4 is sufficient to promote Eomes expression and aspects of the CD8^+^ ILL phenotype in CD8SP thymocytes.

**Figure 3:**
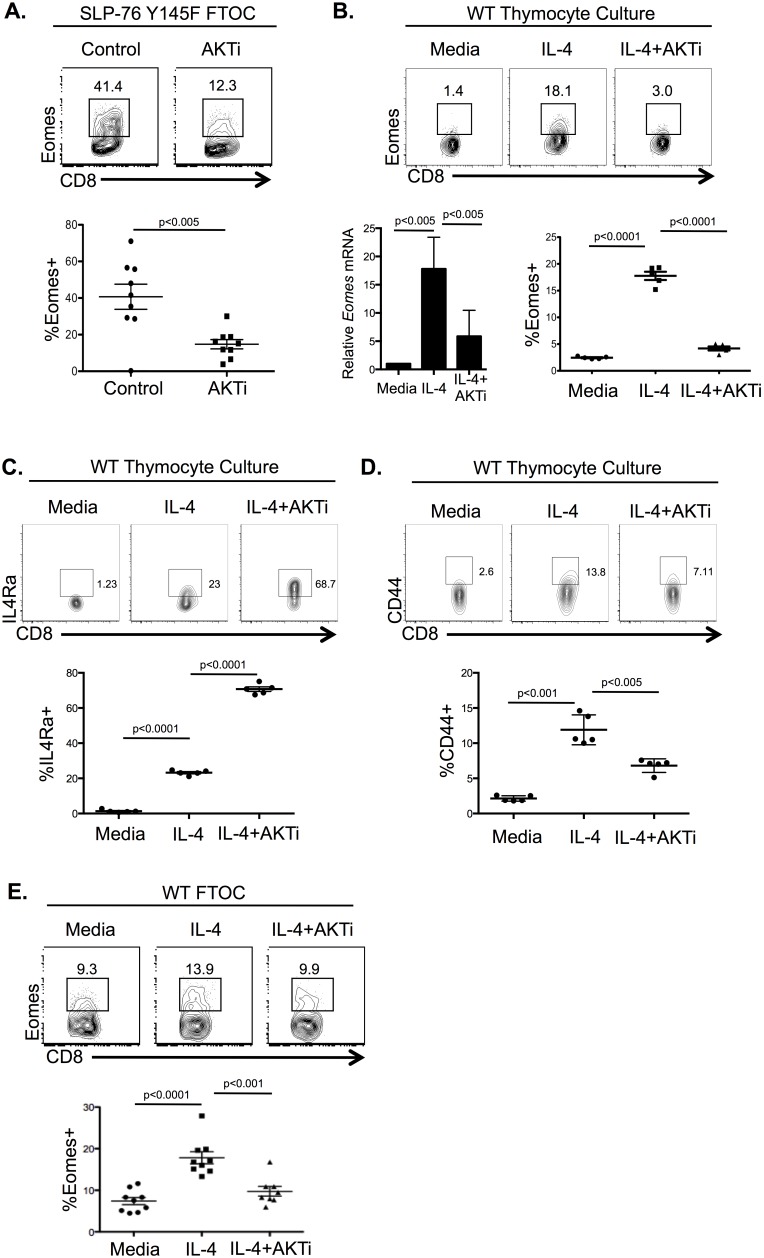
Akt is required for IL-4 regulation of Eomes in CD8SP thymocytes. A) *Top*, Flow cytometric analysis of intracellular Eomes expression in CD8SP thymocytes from d8 SLP-76 Y145F fetal thymi after culture in the absence or presence of AKTi-1/2 (5 µM) for the last 48 h. *Bottom*, Percentage of Eomes^+^ CD8SP among total CD8SP thymocytes in the indicated populations (n = 9, 2 independent experiments). B) *Top*, Flow cytometric analysis of intracellular Eomes expression in WT TCRβ^+^ CD8SP thymocytes after culture in the indicated conditions. *Bottom left*, Relative *Eomes* expression from cDNA of sorted CD8SP WT thymocytes after cultured in the indicated conditions, set relative to matched cells cultured in media alone. *Bottom right*, Percentage of Eomes^+^ cells among total CD8SP thymocytes (n = 5, 2 independent experiments). C) *Top*, Flow cytometric analysis of IL4Ra on CD8SP cells from WT thymocytes cultured as above. *Bottom*, percentage of IL4Ra^+^ cells among total CD8SP thymocytes in indicated conditions (n = 5/condition, 2 independent experiments). D) Flow cytometric analysis of surface CD44 on CD8SP thymocytes treated under indicated conditions as above. *Right*, percentage of CD44^+^ cells among total CD8SP thymocytes (n = 5/condition, 2 independent experiments). E) *Top*, Flow cytometric analysis of intracellular Eomes expression in TCRβ^+^ CD8SP thymocytes from WT fetal thymi after culture for 8d in the indicated conditions. *Bottom*, Percentage of Eomes^+^ cells among total CD8SP population (n = 8–9 per group, 2 independent experiments). Numbers in flow plots represent the percent of the gated population. Graphs show the average percentage of the indicated population and standard error of mean. Statistical significance calculated using Student’s t-test.

### STAT6 and Akt are required for IL-4 induction of Eomes in CD8SP thymocytes

STAT6 and Akt are activated downstream of IL-4 in mature CD8^+^ T cells [32], [33]. Therefore, to investigate the required signal transduction pathways involved in IL-4-directed CD8^+^ ILL development, we examined the basal activation status of these molecules in CD8^+^ ILLs. For these studies, we initially utilized SLP-76 Y145F mice, due to the abundant population of CD8^+^ ILLs present in these mice [12]. Using phospho-flow cytometry, we observed elevated expression of phospho-STAT6 and phospho-Akt in CD8^+^ ILLs *ex vivo* compared to conventional CD8SP thymocytes (Figure 1E). To ensure that these findings were not due to the signaling abnormalities associated with the SLP-76 mutation, we also examined WT CD8SP thymocytes cultured with IL-4. As shown (Figure 1F), we observed higher levels of Akt and STAT6 phosphorylation in this population suggesting that IL-4 activates both pathways in WT CD8SP thymocytes.

To determine if Akt and STAT6 are required for IL-4 to induce Eomes expression in CD8SP thymocytes, we used genetic deficiency or pharmacologic inhibition to block these two proposed arms of IL-4 signaling in CD8SP thymocytes. To examine the role of STAT6 in IL-4 regulation of Eomes in CD8SP thymocytes, STAT6^−/−^ and WT thymocytes were cultured in the absence or presence of IL-4. IL-4 did not significantly promote Eomes transcription or protein expression in CD8SP thymocytes from STAT6^−/−^ mice (Figure 2A–B), indicating that STAT6 is necessary for Eomes induction in response to IL-4. Moreover, in the absence of STAT6, IL-4 was unable to upregulate IL4Ra expression (Figure 2C) but did support partial induction of CD44 (Figure 2D) in CD8SP thymocytes.

Akt signaling is required in the early stages of thymocyte development, as mice doubly deficient in Akt1 and Akt2 have dramatically reduced numbers of double-positive (DP) thymocytes [34], [35]. Therefore, to investigate the role of Akt in CD8^+^ ILL development, we circumvented this early developmental block by administration of the pharmacological inhibitor AKTi-1/2 to mature FTOC. Fetal thymi were cultured initially without inhibitors to allow for normal development of DP thymocytes and then AKTi-1/2 was added to block Akt signaling for the last 48 hours of culture. Using fetal thymi from SLP-76 Y145F mice, we found that Akt inhibition significantly blocked CD8^+^ ILL development (Figure 3A). However, the development of PLZF^+^ cells was also affected with this treatment (data not shown), raising the question of whether Akt signaling was required within the developing CD8SP thymocyte or if these data reflected a requirement for Akt in the development of the IL-4-producing PLZF^+^ population responsible for CD8^+^ ILL development. To address this possibility, we provided exogenous IL-4 to WT thymocytes in the absence or presence of the Akt inhibitor. Akt inhibition abrogated the ability of IL-4 to upregulate Eomes in CD8SP thymocytes (Figure 3B), as measured both by relative mRNA levels and protein expression. Conversely, Akt inhibition did not inhibit IL-4 induced IL4Ra expression; in fact, in the presence of Akt inhibition IL4Ra expression was augmented (Figure 3C). Akt inhibition also partially blocked the IL-4 mediated induction of CD44 on CD8SP thymocytes (Figure 3D).

To further test if Akt inhibition affected IL-4 induced Eomes expression in developing WT CD8SP thymocytes, we turned to FTOC and found that Akt inhibition blocked the development of CD8^+^ ILLs in WT fetal thymi cultured with IL-4 (Figure 3E). Taken together, these data suggest Akt is required for IL-4 induction of Eomes expression and optimal CD44 expression in CD8SP thymocytes. Interestingly, these data also provide evidence that STAT6 but not Akt1/2 is required for the upregulation of IL4Ra in response to IL-4 and that Akt signaling may negatively regulate IL4Ra expression under these conditions since IL-4Ra expression was enhanced by Akt inhibition.

**Figure 4:**
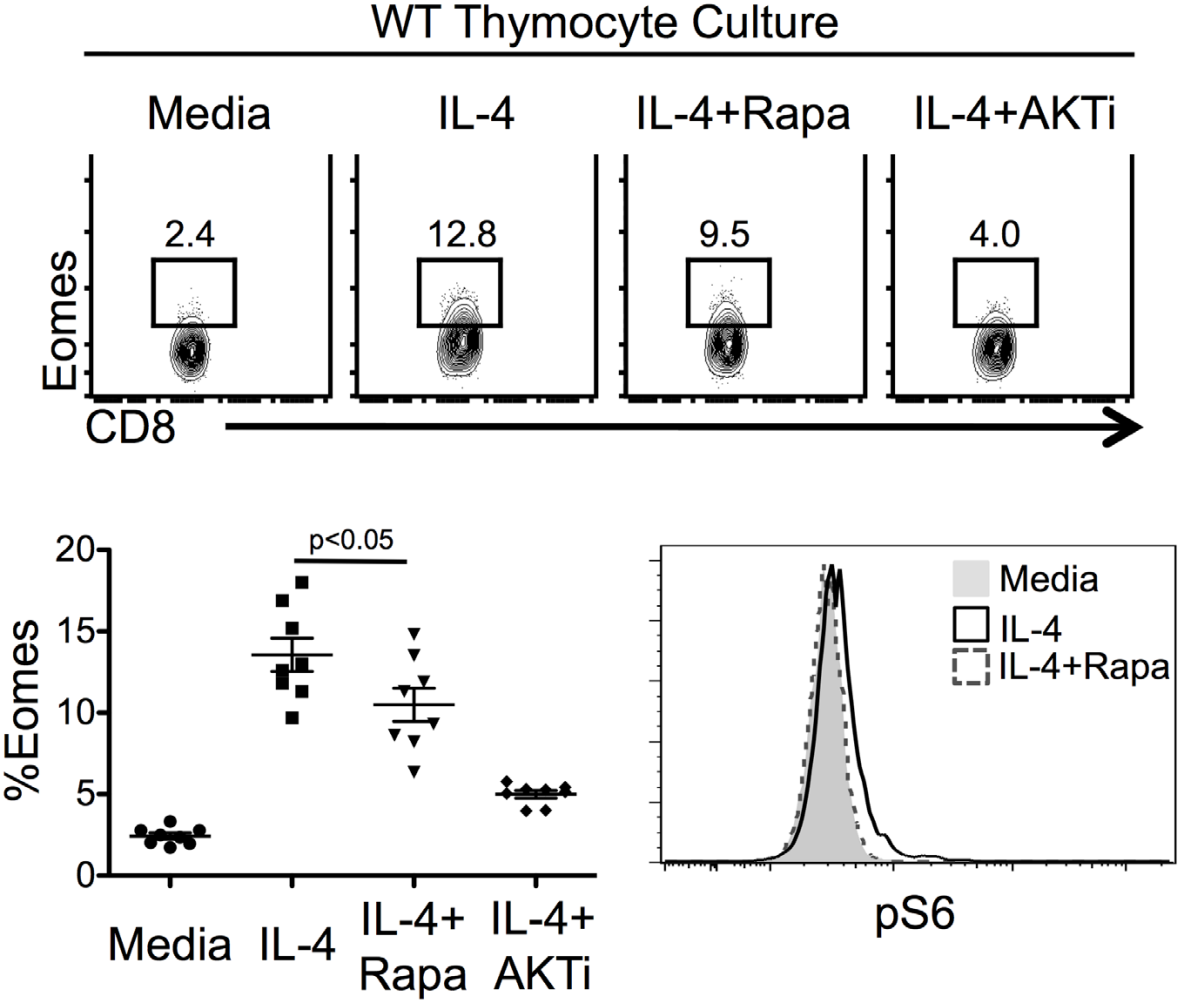
mTORC1 is partially required for IL-4 regulation of Eomes in CD8SP thymocytes. *Top*, Flow cytometric analysis of intracellular Eomes expression in WT TCRβ^+^ CD8P thymocytes after culture in the indicated conditions for 20 h. *Bottom left*, Percentage of Eomes^+^ cells among total CD8SP thymocytes (n = 8, 3 independent experiments). *Bottom right*, Flow cytometric analysis of intracellular phopho-S6 expression in WT CD8SP thymocytes after culture in indicated conditions for 20 h, representative of n = 5 per group, 2 independent experiments. Numbers in flow plots represent the percent of the gated population. Graphs show the average percentage of the indicated population and standard error of mean. Statistical significance calculated one-way ANOVA with Tukey’s multiple comparison post-test.

Akt signals to multiple downstream targets, including mammalian target of rapamycin complex 1 (mTORC1). To determine if mTORC1 is required for the IL-4 induction of Eomes, we treated WT thymocytes with IL-4 in the absence or presence of rapamycin, which inhibits mTOR signaling. While rapamycin treatment was sufficient to inhibit the phosphorylation of S6 (an mTORC1 target) in CD8SP thymocytes, it only partially blocked IL-4 induction of Eomes in these cells. This outcome was in contrast to the near complete inhibition seen following treatment with AKTi-1/2 (Figure 4). These data suggest the mTORC1 pathway is important for optimal Eomes expression in CD8^+^ ILLs, but that Akt also utilizes other, mTORC1 independent signals to regulate expression of this transcription factor.

### IL-4 upregulates Eomes in memory CD8^+^ T cells

**Figure 5:**
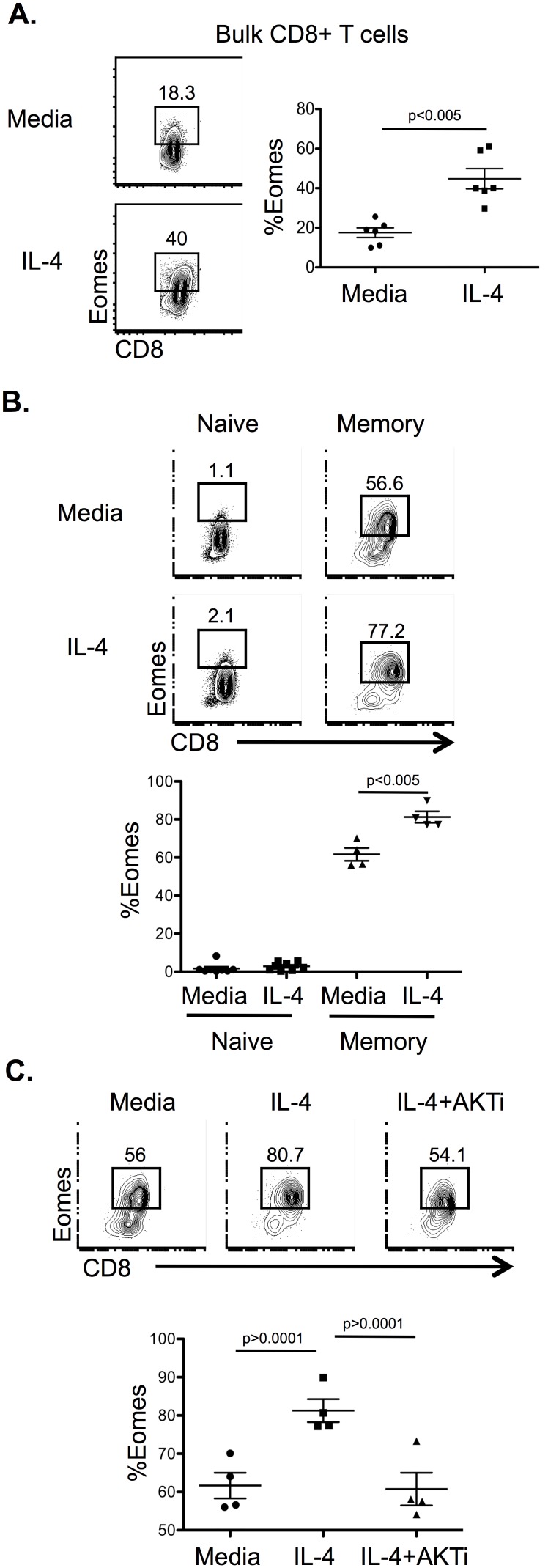
IL-4 upregulates Eomes in memory CD8^+^ T cells in an Akt-dependent manner. A) *Right*, Flow cytometric analysis of Eomes expression in WT peripheral CD8^+^ T cells. *Left*, Percentage of Eomes^+^ cells among total live CD8^+^ T cells after culture for 20 h in the indicated conditions (n = 6, 3 independent experiments). B) *Top*, Flow cytometric analysis of Eomes expression in live TCRβ^+^ CD8^+^ T cells from WT naïve (CD44^−^CD62L^+^) and memory (CD44^+^CD62L^+^) CD8^+^ splenocytes cultured in indicated conditions for 20 h. *Bottom*, Percentage of Eomes^+^ cells among total live CD8^+^ T cells (n = 8 for naïve CD8^+^ cells, 3 independent experiments; n = 4 for memory CD8^+^ T cells, 2 independent experiments). C) *Top*, Flow cytometric analysis of intracellular Eomes expression in WT memory CD8^+^ T cells treated for 20 h in indicated conditions. *Bottom*, Percentage of Eomes^+^ cells among live memory CD8^+^ T cells (n = 4, 2 independent experiments). Live lymphocyte gate was determined by forward and side scatter gating. Numbers in flow plots represent the percent of the gated population. Graphs show the average percentage of the indicated population and standard error of mean. Statistical significance calculated using Student’s t-test (A, B) or one-way ANOVA with Tukey’s multiple comparison post-test (C).

Since IL-4 directs CD8^+^ ILL cell development in the thymus, we posited that IL-4 may also regulate cell fate and function of peripheral CD8^+^ T cells through its regulation of Eomes. When WT peripheral T cells were cultured with IL-4, Eomes expression was induced in CD8^+^ T cells (Figure 5A). Peripheral CD8^+^ T cells are a heterogeneous population containing both naïve and memory subsets. Therefore, to identify the IL-4 responsive population, we sorted naïve (CD44^−^CD62L^+^) and memory (CD44^+^) CD8^+^ T cells and cultured them in the absence or presence of IL-4. In memory CD8^+^ T cells but not in naïve CD8^+^ T cells, IL-4 promoted significant Eomes expression (Figure 5B). This effect correlated with IL4Ra expression (data not shown). Similar to WT CD8SP thymocytes, in memory CD8^+^ T cells, Akt inhibition blocked the IL-4 induction of Eomes expression (Figure 5C).

### Differential modulation of IFNγ and Eomes by IL-4 and TCR in naïve CD8^+^ T cells

**Figure 6:**
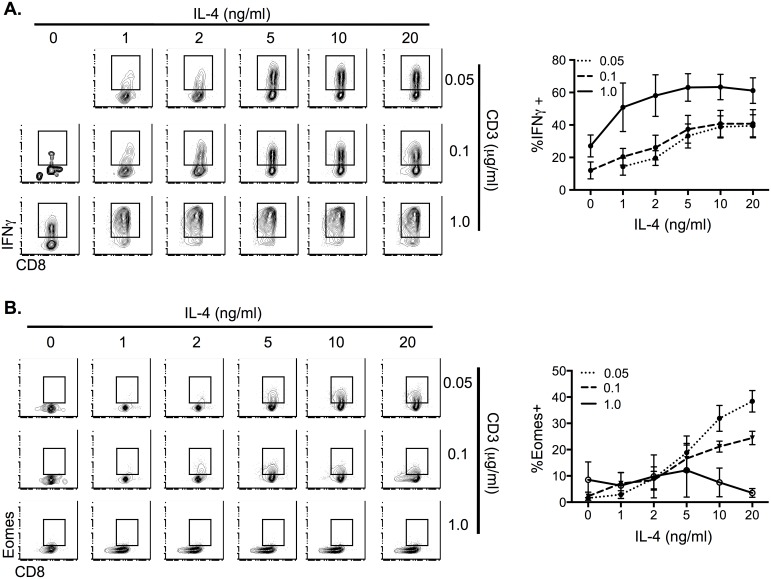
IL-4 sensitizes naïve CD8^+^ T cells to TCR stimulation and promotes Eomes expression during low TCR activation. A) *Left*, Flow cytometric analysis of intracellular IFNγ expression in naïve CD8^+^ T cells activated for 3d with anti-CD3/CD28 with varying concentrations of IL-4, rested for 2d and then re-stimulated with PMA and ionomycin. *Right*, Percentage of IFNγ^+^ T cells among total live cells after stimulation in indicated conditions. B) *Left*, Flow cytometric detection of intracellular Eomes expression in naïve CD8^+^ T cells activated for 3d with anti-CD3/CD28 with varying concentrations of IL-4 then rested for 2d. *Right*, Percentage of Eomes^+^ cells among total live cells after stimulation in indicated conditions. Data are pooled from five independent experiments. Graphs show the average percentage of the indicated population and standard error of mean.

Given that CD8SP thymocytes upregulate Eomes in response to IL-4 alone but naïve CD8^+^ T cells were significantly less susceptible to Eomes induction, we hypothesized that another signal may be required in addition to IL-4 to promote robust Eomes expression in naïve peripheral CD8^+^ T cells. Since both developing CD8SP thymocytes and memory CD8^+^T cells may have experienced recent TCR stimuli during either development or differentiation, we reasoned that TCR signaling may synergize with IL-4 to upregulate Eomes in naïve CD8^+^ T cells. To determine if TCR stimulus cooperates with IL-4 in naïve CD8^+^ T cells, we activated these cells with various doses of anti-CD3 with anti-CD28 in a range of IL-4 concentrations for 3d, followed by a 2d rest in the presence of low dose IL-2. In naïve CD8^+^ T cells, IL-4 potentiated IFNγ production in a dose-dependent manner across all concentrations of TCR stimulus (Figure 6A), suggesting that IL-4 enhances CD8^+^ T cell effector function after T cell activation. However, IL-4 only promoted Eomes expression in CD8^+^ T cells activated with low doses of TCR (Figure 6B). These data suggest IL-4 enhances IFNγ production at all doses of TCR activation, but that it only cooperates with low dose TCR stimulus to induce Eomes expression during CD8^+^ T cell activation and that high dose TCR stimulus blocks the IL-4 effect on Eomes expression.

## Discussion

In this study, we examined the cellular and biochemical requirements by which IL-4 regulates CD8SP thymocyte development and peripheral CD8^+^ T cell function. We show that IL-4 induction of Eomes and several CD8^+^ ILL markers require STAT6 and Akt signaling. In addition, we dissected the individual contributions STAT6 and Akt pathways play in the IL-4 driven expression of CD8^+^ ILL markers, including IL4Ra and CD44 expression on CD8SP thymocytes. In peripheral cells, we found that IL-4 cooperates with TCR stimulation to enhance IFNγ production by CD8^+^ T cells and promotes Eomes expression in CD8^+^ T cells activated with low dose TCR plus IL-4.

Analysis of the signaling pathways required for IL-4 induction of Eomes and CD8^+^ ILL development demonstrate that Akt and STAT6 are essential. STAT6 is the canonical signaling molecule associated with many IL-4 responses [36], [37]. We show here that phosphorylation of STAT6 is induced in CD8SP thymocytes following stimulation with IL-4 and that STAT6 phosphorylation is increased in CD8^+^ ILLs compared to CD8^+^ non-ILLs. Moreover, using STAT6-deficient mice, we demonstrate that STAT6 is required for IL-4 induction of Eomes transcription and protein expression in CD8SP thymocytes, as well as induction of IL4Ra expression. One possible explanation for the inability of IL-4 to upregulate Eomes expression in STAT6-deficient CD8SP thymocytes could be insufficient IL4Ra expression on these cells and thus lack of sustained Akt signaling. However, IL-4 continues to induce pAkt in STAT6-deficient CD8SP thymocytes in response to IL-4 suggesting Akt signaling is intact (unpublished results, S.A.C.), making this possibility less likely. In addition, STAT6 loss only partially abrogates IL-4 driven upregulation of CD44 on CD8SP thymocytes, further providing evidence that some component(s) of IL-4 downstream signaling remains active in these cells. These data are consistent with the requirement for STAT6 in the development of CD8^+^ ILLs that arise in a murine model in which thymocytes are selected by MHC class II-expressing thymocytes [38]. How STAT6 induces Eomes expression in CD8SP thymocytes is unknown, but one possible mechanism could be through direct regulation of *Eomes* transcription, as we demonstrate that IL-4 induction of Eomes message is abrogated in STAT6-deficient CD8SP thymocytes and STAT6 has been shown to bind to the Eomes promoter in mature CD4^+^ T cells [39].

Our data indicate that Akt is a major regulator of IL-4-induced Eomes expression and CD8^+^ ILL thymocyte development, since Akt inhibition abrogates the ability of IL-4 to induce Eomes and partially blocks upregulation of CD44 on CD8SP thymocytes *in vitro*, as well as the ability to develop Eomes^+^ CD8SP thymocytes in FTOC. The ability both of Akt inhibitors and STAT6 deficiency to block Eomes induction suggests that cooperation between these two signaling pathways is required for IL-4 induced Eomes expression in CD8SP thymocytes. In contrast, since CD44 expression is not completely lost in the context of either STAT6 deficiency or Akt inhibition, it appears that STAT6 and Akt can independently regulate IL-4 mediated CD44 expression. Interestingly, Akt inhibition strongly enhanced IL4Ra expression in IL-4 treated CD8SP thymocytes. This finding was in contrast to the near complete requirement of STAT6 for IL4Ra expression. Thus, these data reveal that IL4Ra is reciprocally regulated by STAT6 and Akt following IL-4 receptor engagement, and suggest that Akt signaling is involved in negative regulation of this receptor.

Investigating potential pathways downstream of IL-4 induced Akt activation, we found that mTORC1, a well described mediator of Akt signaling, also controls, though is not fully required for, IL-4-driven Eomes expression. Another possible downstream effector of Akt signaling is Foxo1, which is negatively regulated by Akt phosphorylation. However, Foxo1 has been shown to bind to the Eomes promoter and enhance its expression in peripheral CD8^+^ T cells [40], which would be counter to our observations here in which Akt activity promotes Eomes expression. GSK3α/β is another Akt target that is phosphorylated and inactivated by Akt. Its inactivation allows for the association of β-catenin with TCF1, which promotes transcription of TCF1 target genes. TCF1 has been demonstrated to directly associate with the Eomes locus and be required for its expression in CD8^+^ T cells [41], but our preliminary observations suggest that TCF1 is not required for IL-4-driven Eomes expression. Studies aimed to identify the Akt targets that mediate IL-4-driven Eomes expression are ongoing.

There are different requirements for IL-4 induction of Eomes in thymocytes and peripheral CD8^+^ T cells, since IL-4 alone is insufficient to drive Eomes protein expression in naïve peripheral CD8^+^ T cells. We demonstrate that naïve CD8^+^ T cells can upregulate Eomes expression in response to IL-4 when there is a concomitant TCR signal. Interestingly, this effect is only seen with low but not high dose TCR stimulus. These data are consistent with the finding that dampening TCR signal transduction via inhibition of Itk, a proximal signaling kinase required for optimal TCR signal transduction, and subsequent downregulation of IRF4-mediated repression of Eomes results in increased susceptibility of naïve T cells to Eomes induction following IL-4 plus TCR stimulation [28]. Given that IL-4 promotes Eomes expression in the setting of attenuated TCR signaling, but not during robust TCR activation, suggests that cytokine signaling pathways may differentially alter cell fate depending on the strength and/or abundance of TCR signals. This finding may be relevant in situations where antigen is limited or in T cells with lower TCR affinity, directing them to a different cell fate based on relative Eomes expression.

In our experiments, we also noted that IL-4 promoted IFNγ production in naïve CD8^+^ T cells activated with a wide range of TCR stimulus doses. These data are in contrast to findings in CD4^+^ T_H_2 differentiation in which IL-4 inhibits IFNγ production [5]. Though Eomes is known to regulate IFNγ production [17], this effect does not require robust Eomes expression, since IFNγ production occurred at all TCR stimulus doses and did not correlate with Eomes protein levels. Although the mechanism by which IL-4 enhances IFNγ production during TCR activation is unknown, the mitogen-activated protein kinase and phosphoinositide 3-kinase pathways have been implicated [33]. Moreover, a recent report demonstrating a role for GATA3 in TCR and cytokine-induced IFNγ production in CD8^+^ T cells [42] and the fact that GATA3 expression can be induced by both TCR and IL-4 signaling [5], [42], [43] provides support for this transcription factor as a potential candidate [42]. How thymically-derived CD8^+^ ILLs or peripheral T cells exposed to IL-4 in concert with low TCR triggering, contribute to T cell immune responses *in vivo* has not been fully defined. The data presented here provide insight on the molecular pathways regulating changes in transcription and protein expression upon exposure of CD8^+^ T cells to IL-4, and highlight how IL-4 may affect CD8^+^ T cell differentiation and function under varying conditions of TCR signal strength. Further understanding of how signaling pathways emanating from the TCR and IL-4R impact different aspects of CD8^+^ T cell differentiation may prove beneficial for modulating immune responses.

## Supporting Information

Figure S1 **IL-4 promotes Eomes expression in WT CD8SP thymocytes.** A) Flow cytometric analysis of intracellular Eomes expression with rat IgG2a, kappa isotype control in CD8SP cells from WT thymocytes cultured in the absence or presence of IL-4 (20 ng/ml) for 20 h. Plots are gated on live, TCRb^hi^ CD8SP lymphocytes. Data are representative of two mice.
(TIFF)
