# Supplementary figures and images for: Interleukin-4 Regulates Eomesodermin in CD8+ T Cell Development and Differentiation
Source: PLoS One. 2014 Sep 10;9(9):e106659. doi: 10.1371/journal.pone.0106659 (PMC4160212; doi:10.1371/journal.pone.0106659)

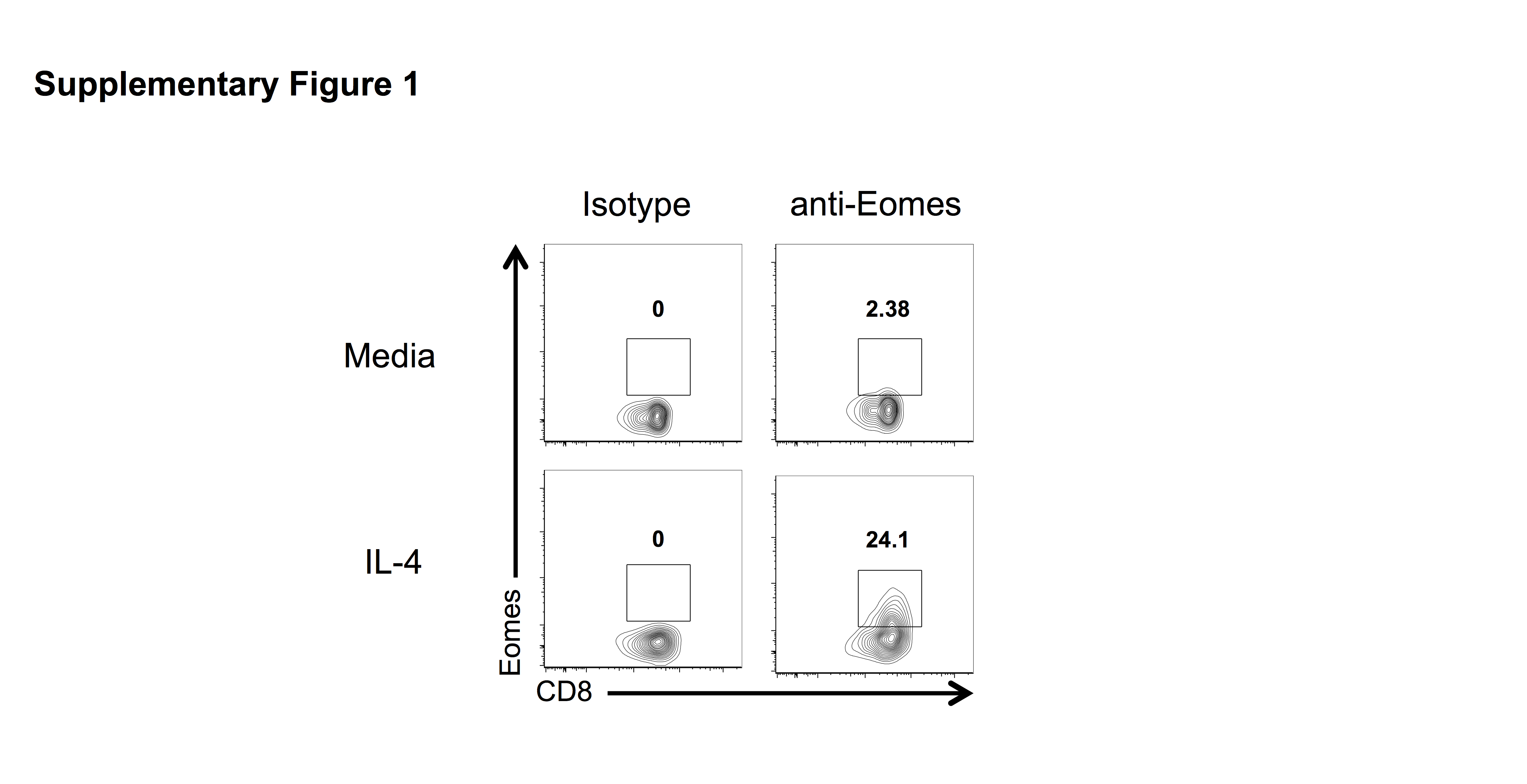

Supplement: Figure S1 — IL-4 promotes Eomes expression in WT CD8SP thymocytes. A) Flow cytometric analysis of intracellular Eomes expression with rat IgG2a, kappa isotype control in CD8SP cells from WT thymocytes cultured in the absence or presence of IL-4 (20 ng/ml) for 20 h. Plots are gated on live, TCRbhi CD8SP lymphocytes. Data are representative of two mice. (TIFF) [file pone.0106659.s001.tiff]
